# Second- and Further-Line Therapy with Erlotinib in Patients with Advanced Non-Small-Cell Lung Cancer in Daily Clinical Practice

**DOI:** 10.1155/2014/987150

**Published:** 2014-06-17

**Authors:** Josephine Krainhöfer, Mario Walther, Matthias Steinert, Angelika Reissig

**Affiliations:** ^1^Department of Internal Medicine I, Pneumology & Allergology, Medical Clinic I, Jena University Hospital, Friedrich Schiller University, Erlanger Allee 101, 07740 Jena, Germany; ^2^Institute of Medical Statistics, Computer Sciences and Documentation, Jena University Hospital, Friedrich Schiller University, Erlanger Allee 101, 07740 Jena, Germany; ^3^Department of Cardiothoracic Surgery, Jena University Hospital, Erlanger Allee 101, 07740 Jena, Germany

## Abstract

*Introduction*. The aim of this retrospective study was to examine effect of erlotinib in patients with advanced non-small cell lung cancer (NSCLC) in second-line and further therapy in daily clinical practice. *Methods*. Patients with histologically or cytologically proven NSCLC (*n* = 84) treated with erlotinib in second-line (*n* = 34), third-line (*n* = 36), and more-line therapy (*n* = 14) were examined for progression-free survival (PFS), overall survival (OS), disease control rate (DCR), duration of therapy, and adverse effects. *Results*. Median PFS of all lines was 83 days (CI 70.0–96.0), OS was 7 months (CI 4.7–9.3), DCR was 66.2% (CI 55–77%), and 1-year survival rate was 33% (CI 22–43%), with no significant difference between therapy lines. Median duration of treatment was 76 days (IQR 39–139.5). Patients with epidermal growth factor receptor mutation (EGFR-M) reached the highest PFS (204 days), as did patients with good performance status (ECOG 0-1: 94 versus ECOG 2-3: 65 days, *P* = 0.035). Patients with EGFR-M also revealed a DCR of 100%. The most frequent side effects were rash (69%) and diarrhoea (41%), without any significant difference between therapy lines. In 24 patients, the treatment dose was reduced and in 18, the therapy was paused. *Conclusion*. Erlotinib works in all therapy lines without any significant differences in efficacy and side effects.

## 1. Introduction

Even though smoking, the main risk factor for lung cancer, can be avoided, the incidence of lung cancer is still increasing. Through all stages, the 5-year survival rate is only about 16% for males and 19% for females. Also, only 2% of the 40% of patients that are diagnosed in stage IV are still alive after 5 years [1–3].

Erlotinib is an inhibitor of epidermal growth factor receptor (EGFR) that blocks the binding site for intracellular ATP [4]. Therefore, ligands can no longer activate the receptor, because signalling pathways are initiated through the phosphorylation of tyrosine with ATP [5]. Active EGFR increases proliferation, cell motility, and angiogenesis and induces antiapoptotic proteins [6–9]. As a consequence, erlotinib is supposed to have the opposite effect on tumour cells [10]. It is approved in the treatment of non-small-cell lung cancer (NSCLC) in the first-line when patients are EGFR mutation-positive and in second-line and more-line therapy regardless of mutation status [11]. An essential advantage of erlotinib is the simple form of daily oral application, which is well tolerated by patients.

In a phase IV study about efficacy and safety of erlotinib therapy, the disease control rate (DCR) was 69%, mostly stable diseases (SD) (55%) [12]. Progression-free survival (PFS) and overall survival (OS) were 3.3 and 7.9 months, respectively. In a subgroup of patients, the influence of EGFR mutation was examined. About 50% of subjects with a mutation had a response compared to 3% with wild type receptor. Also, 65% of patients suffered from rash and 10% from diarrhoea, and therapy had to be discontinued in 5% due to side effects. Further side effects were emesis, vomiting, stomatitis, abdominal pain, fatigue, dyspnoea, cough, anorexia, infections, conjunctivitis, and keratoconjunctivitis sicca. Mouth ulcers, paronychia, alopecia, dysgeusia, and increased liver parameters and bilirubin were seldom observed [12, 13].

Erlotinib is well examined in first-line therapy [14] and second-line therapy [15]. Therefore, the topic of this study was to assess the impact and side effects of erlotinib in second-line and more-line therapy in daily clinical practice. Furthermore, we had a closer look at the differences between the different lines.

## 2. Patients and Methods

This is an open-label, monocentric, retrospective, observational study. The local ethics committee approved the study (number 3570-09/12).

Patients aged over 18 years with advanced histologically or cytologically confirmed NSCLC stage IIIB and stage IV (according to the seventh TNM classification [16]) and at least one accessible tumour lesion, who had received at least one line of therapy, were enrolled. After explaining the therapeutic options to the patients, of either continuing intravenous chemotherapy or receiving erlotinib, all patients that chose erlotinib in second-line and more-line therapy were analysed. They are defining the study population. Normal renal and hepatic function (not exceeding twice the normal range), as well as sufficient haematological function (white blood cells ≥ 3 × 10^9^/litre and/or thrombocytes ≥ 100 × 10^9^/litre), were required. Combined radiochemotherapy was regarded as the first systemic therapy line.

Exclusion criteria were stages I, II, and IIIA, first-line therapy with erlotinib, or inappropriate renal and liver organ function.

Each patient was instructed to take the drug at the same time once a day, with a gap between meals. Usually, the starting dose was 150 mg per day, but patients in a worse condition could receive a reduced dose from the start. Each patient was advised to apply skin protection to avoid rashes and how to prepare food in case of diarrhoea. If worse adverse effects (grade III or IV) occurred, treatment was interrupted until recovery. Afterwards, therapy was continued as before or with a dose reduction or even finished in some cases, depending on the individual scenario. Symptomatic therapy included loperamide for diarrhoea and cortisone- and/or antibiotic-containing ointments for rashes.

Patients were examined once a month with regard to side effects (classification: Cancer Therapy Evaluation Program, Common Terminology Criteria for Adverse Events, Version 4.0 [17]) or indicators of progression. In case of clinical deterioration or pathological physical examination, further diagnostic assessment was performed immediately. Usually, tumour size was assessed by computed tomography (CT) or positron emission tomography/CT (PET/CT) every three months.

Patients were assessed for histology, smoking status, tumour stage, and performance status when erlotinib was introduced. Moreover, the duration of erlotinib therapy, number of previous and following therapies, and side effects of erlotinib were evaluated.

*Statistical Analysis*. The primary endpoint PFS was calculated from the beginning of medication with erlotinib until progress was observed or patient's death. Secondary end points were OS, defined from the start of erlotinib treatment until death, and response rate, evaluated with regard to the response evaluation criteria in solid tumors (RECIST) version 1.0 [18].

Qualitative data are given as counts and percentages. Quantitative data are characterised by median and interquartile range (IQR). To compare best response and disease control rate (DCR) between different therapy lines, the exact Chi² test was used.

For progression-free survival (PFS) as well as OS overall survival, the Kaplan-Meier method was used to obtain estimates for median survival together with the 95% confidence interval (CI). The log rank test was used for comparison of survival curves of patients with different characteristics. The level of significance was set at 0.05. Statistical analysis was conducted using SPSS 21.0 for Windows. Data cut-off was the June 30, 2013.

## 3. Results

### 3.1. Baseline Characteristics

**Figure 1:**
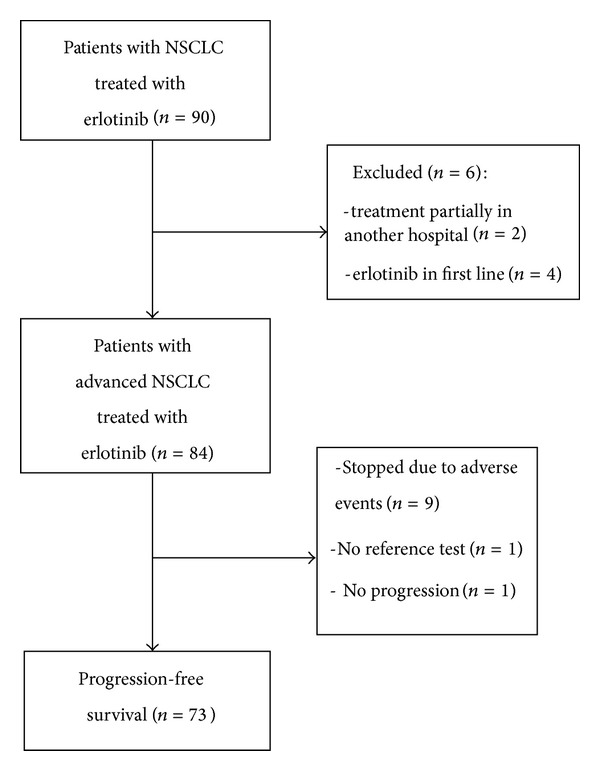
Flow chart.

In the Department of Internal Medicine I, Pneumology and Allergology, Jena University Hospital, 90 patients with NSCLC were treated with erlotinib from October 2005 to December 2012. Of these patients, 84 met the inclusion criteria and were included (Figure 1). The baseline characteristics of the patient cohort are described in Table 1. The patients were predominantly male (86.9%) in stage IV (97.6%) with Eastern Cooperative Oncology Group (ECOG) performance status (PS) 1 (47.6%) or 2 (41.7%). The main histotypes were squamous cell carcinoma (56.0%) and adenocarcinoma (23.8%). The median age at the start of erlotinib treatment was 70 years (IQR 63–73, range 50–84). Current or former smokers made up 76.2% of the study population. The EGFR mutation status was known for 24 (28.6%) of the patients; four of them (16.7%) were mutation-positive. In one patient, the mutation status was not determinable. The ethnicity was Caucasian.

Before treatment with erlotinib, patients were exposed to 14 different kinds of mono- and double-combination chemotherapies; in 14.3% of patients, combined radiochemotherapy was performed. Most of the therapies contained platinum (98.8%). The most frequent therapies were paclitaxel combined with carboplatin (*n* = 72; 85.7%), docetaxel (*n* = 31; 36.9%), and vinorelbine combined with carboplatin (*n* = 13; 15.5%). Generally, patients received two therapy lines before erlotinib; 34 patients (40.5%) were given erlotinib as a second-line, 36 (42.9%) as a third-line, and 14 (16.7%) received erlotinib in fourth-line or more-line therapy (12 in fourth-line, one in fifth-line, and one in sixth-line) (Table 1).

### 3.2. Erlotinib Therapy

The median* duration* of treatment with erlotinib was 76 days (IQR 39–139.5), ranging from one to 851 days.

**Figure 2:**
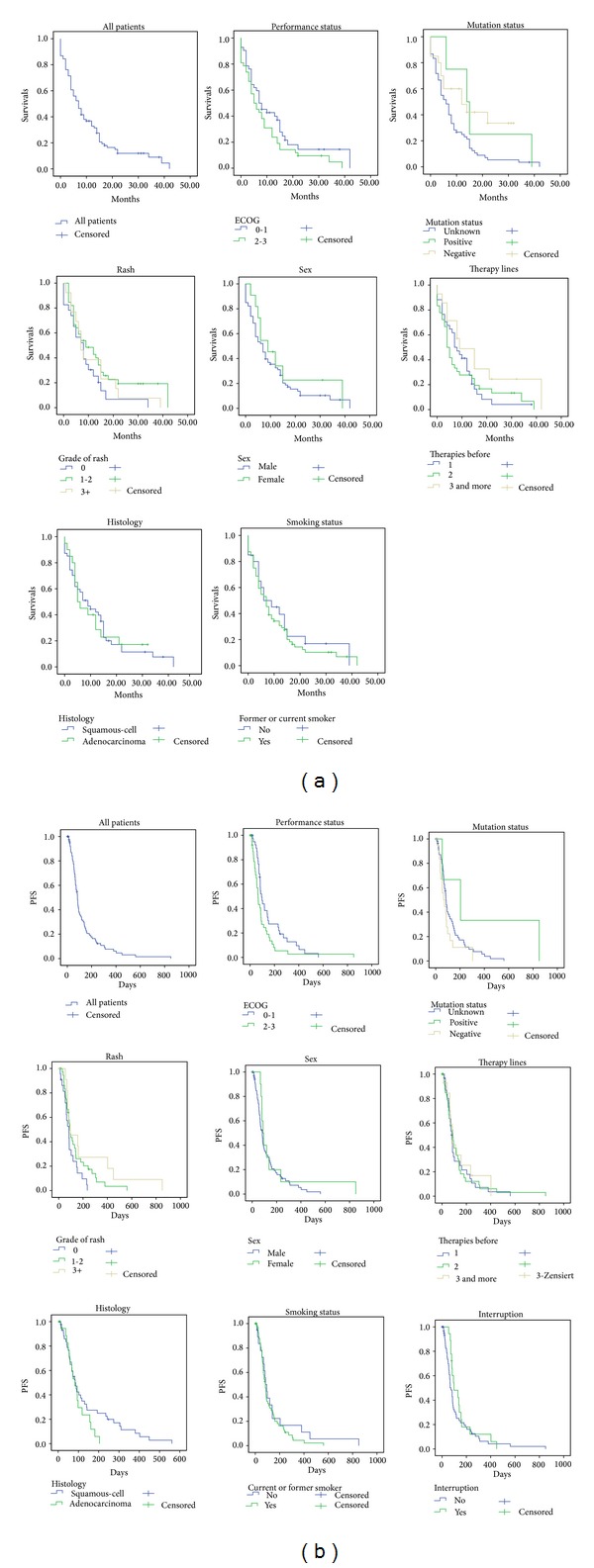
Kaplan-Meier curves for overall survival (OS) (a) and progression-free survival (PFS) (b) according to ECOG performance status, EGFR-mutation status, grade of rash, sex, number of therapy lines, histology, smoking status and treatment interruption (only in (b)).

The median* OS* of all patients was 7 months (CI 4.7–9.3). Concerning patient's characteristics (Table 1), no significant difference in OS was observed in any subgroup. Nevertheless, in males (7 versus 9 months in females, *P* = 0.38), patients with adenocarcinoma (5 versus 9 months in squamous cell carcinoma, *P* = 0.99) and patients with worse ECOG performance status (ECOG 2-3: 5 months versus ECOG 0-1: 7 months, *P* = 0.127) had a shorter median OS. Referring to skin toxicity, patients without a rash had a median OS of 8 months, those with a mild rash (grades 1-2) had a median OS of 9 months, and in cases of severe rash (grades 3-4), the median OS was 7 months (*P* = 0.217). With respect to the therapy lines, erlotinib in second-line had a median OS of 7 months and in third-line 4 months and for fourth-line and more-line patients, this was 9 months (*P* = 0.178). Smoking history had no significant influence on median OS (smokers 7 months, never smokers 6 months, *P* = 0.513). Concerning mutation status, patients with EGFR mutations had a median OS of 14 months (CI 5.1–22.9), while patients who were EGFR-M negative had an OS of 12 months (CI 0.6–23.4) (the log rank test was not conducted because of the small number of mutation-positive patients (*n* = 4)) (Table 2 and Figure 2(a)).

With a closer look at the Kaplan-Meier curves of median OS, we determined one-year survival of the different therapy lines; no significant difference was observed (*P* = 0.178). Between all lines, 33% of patients were alive after 12 months, including 49% of those receiving fourth-line and more-line therapy and 31% receiving second-line therapy, and the lowest value of 28% was observed in third-line therapy (Table 2).

The median* PFS* of all patients was 83 days (CI 70.0–96.0). Females (median PFS 89 days versus 83 days in males, *P* = 0.32), never smokers (median PFS 94 versus 83 days in smokers, *P* = 0.401), patients with a rash (grades 1-2: 91 days, grades 3-4: 94 days, and no rash: 83 days, *P* = 0.065), and patients with treatment interruption due to side effects (median PFS 98 days versus 73 days in patients without interruption, *P* = 0.259) appeared to assume a longer median PFS. Regarding therapy lines, third-line patients had the longest median PFS (89 days versus 80 days in second-line versus 84 days in fourth-line and more-line, *P* = 0.886), but the result was not statistically significant. EGFR-M positive patients had an exceedingly longer median PFS than mutation-negative patients (204 days (CI 0.0–444.1) versus 73 days (CI 35.9–110.1)). Histology had less of an impact on PFS (adenocarcinoma 83 days, squamous cell carcinoma 84 days, *P* = 0.188). A significantly better PFS was observed in patients with good performance status (ECOG 0-1: 94 days versus ECOG 2-3: 65 days, *P* = 0.035, HR 0.61 (CI 0.38–0.972) for ECOG 0-1 versus ECOG 2-3) (Table 2 and Figure 2(b)).

**Figure 3:**
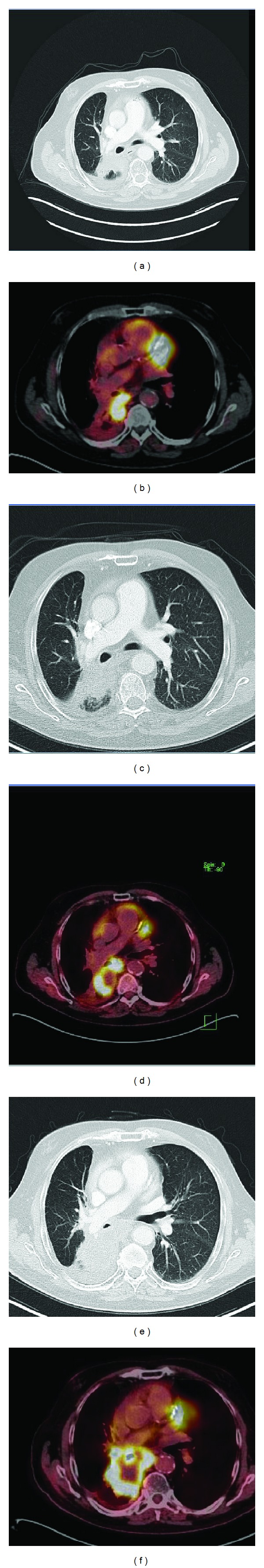
CT and PET/CT scans of a 73-year-old patient with advanced NSCLC before starting erlotinib therapy with 100 mg per day ((a) and (b)). Squamous cell carcinoma is located in the right lower lobe. PET/CT 10 month later ((c) and (d)) shows no significant changes. The size of the tumour is nearly constant. PET/CT 13 months ((e) and (f)) after the start of erlotinib therapy revealed a local progressive disease.

Five patients revealed a remarkably long PFS (12, 13, 14, 18, and 27 months) (Figure 3). The patients were four men and one woman, aged between 71 and 74 years, and all with stage IV disease; two of them received erlotinib as a second-line therapy, two as a third-line, and one as a sixth-line. Four had squamous cell carcinoma and one an additional adenocarcinoma. Three of these patients had a grade 3 rash, while the remaining patients had no side effects higher than grade 2.

Best response to erlotinib was reported for 74 patients. In eight patients, response was not evaluable because treatment was stopped after a few days (1–20 days) due to side effects or worse performance status. A further patient ended the therapy after 4 days due to incompliance, while no reference test was available for another patient.

In the sample group, a DCR of 66.2% (63.5% SD, 2.7% partial response (PR)) was reached. The two patients with a PR had the following characteristics: both were male in stage IV with ECOG 2; one was 76 years old, was in second-line therapy, was a never smoker, was with an adenocarcinoma, was mutation-positive, and had a duration of treatment of 204 days, while the other was 72 years old, was in fourth-line therapy, was a former smoker with squamous cell carcinoma with unknown mutation status, and had a treatment duration of 111 days.

The analysis showed no significant difference of DCR for EGFR mutation-positive patients compared to mutation-negative ones (100% DCR versus 61.1%, *P* = 0.521), probably because of the small patient number.

Other characteristics like sex (DCR males 64.1% versus females 80.0%, *P* = 0.48), histology (DCR squamous cell carcinoma 63.4%, adenocarcinoma 76.5%, and other differentiation 62.5%, *P* = 0.607), rash (DCR no rash 61.9%, grades 1-2 82.9%, and grades 3-4 63.6%, *P* = 0.184), smoking status (DCR former smoker 66.1, never smoker 66.7%, *P* = 1.00), or PS (ECOG 0-1 70.3%, ECOG 2-3 62.2%, *P* = 0.624) did not differ significantly.

In patients with an interruption of treatment due to side effects, a nonsignificant trend for a lower rate of progression was encountered (DCR with interruption 82.4%, without 60.7%, *P* = 0.146). Between different therapy lines, no difference was observed (DCR second-line 58.6%, third-line 69.7%, fourth-line and more-line 75%, *P* = 0.542).

### 3.3. Adverse Events, Dose Reductions, and Treatment Interruptions

Adverse events were unknown in nine cases: seven patients died before their first check-up, one ended therapy for incompliance, and one ended therapy due to a worse performance status.

In one patient, an increase in creatinine level was observed, but as he received potentially nephrotoxic parallel medications, the inducing drug could not be determined.

Overall, 21 different side effects were observed. As known for tyrosine kinase inhibitors (TKI), the most frequent adverse events were any grade of rash (69.3% of the patients) and diarrhoea (41.3%), mostly mild or moderate (rash grades 1-2: 39 patients (52.0%), diarrhoea grades 1-2: 23 (30.6%)). Other common adverse effects were nausea and vomiting, pain, and neurotoxicity.

Regarding patients with any adverse event that was grade 3 or higher, no therapy line had significantly much worse adverse events (all side effects grades 3-4: *P* = 0.695, rash grades 3-4: *P* = 1.00, and diarrhoea grades 3-4: *P* = 0.783), but there appeared to be a trend towards less worse side effects in the third-line group (Table 3).

Treatment dose was reduced in 24 patients (32%); in 11 of them, this was from the onset. Reasons for starting with a lower dose of 100 mg per day were worse performance status in six patients, enduring side effects of prior chemotherapies in four patients, and renal dysfunction in one patient. Due to side effects, the dose was reduced to 100 mg (*n* = 13) or 75 mg (*n* = 1, patient with a starting dose of 100 mg) per day. Reasons for this included rash grade 3 (*n* = 4), rash grade 2 (*n* = 3), diarrhoea grade 3 (*n* = 1), and stomatitis grade 3 (*n* = 1). A further five patients had more than one adverse event, which were described as combined adverse events.

Because of adverse events, erlotinib therapy had to be paused in 24.0% (*n* = 18) of patients. Two of them (11.1%) had a second break. The first break averaged 8.5 days (IQR 7–14.25), ranging from 5 to 29 days. The second breaks were 6 and 8 days. Reasons for pausing were rash (grade 3: *n* = 6, grade 2: *n* = 2), diarrhoea (grade 3: *n* = 2), pain (grade 4: *n* = 2), emesis (grade 3: *n* = 1), stomatitis (grade 2: *n* = 1), and combined side effects (*n* = 4). One of the two patients with two erlotinib breaks had combined side effects of rash, diarrhoea, and pain (all grade 3), whereas the other had rash (grade 3) plus alopecia (grade 2).

### 3.4. Therapies Applied after Erlotinib

For 41 patients (48.8%), erlotinib was the last anticancer medication; 27 patients (32.1%) received one further line, ten (11.9%) were given two more, and six (7.1%) received three more therapy lines. The most frequent therapies were trofosfamide (*n* = 21, 25.0%), docetaxel (*n* = 14, 16.7%), gemcitabine (*n* = 10, 11.9%), and pemetrexed (*n* = 10, 11.9%). Three patients received an irreversible TKI for compassionate use because they had a long-lasting SD under erlotinib (10, 14, and 16 months, resp.).

Taken together, 84 of the included patients received three therapies on average (IQR 3-4), ranging from two-line to seven-line.

## 4. Discussion 

The median OS for all therapy lines was 7 months, which is comparable to the results of 6.7 months which were reported for second-line and third-line therapy in the BR.21 study [15]. Referring to the different therapy lines, no significant differences were observed. Nevertheless, median OS in fourth-line and more-line of nine months was more than twice the median OS in third-line. Interestingly, the DCR as well as the highest one-year survival rate was detected in the fourth-line and more-line therapy group, too. These results might be caused by selection and/or changes in tumour characteristics during several therapy lines which should be confirmed in a larger study.

Median PFS for all lines was 83 days with no significant differences between therapy lines. Mutation-positive patients (*n* = 4) reached the longest median PFS, which was more than twice as high as the median of all patients. This is in good accordance with other studies, where an EGFR mutation was associated with a long-lasting response [13] or high PFS in first-line therapy [14]. However, median PFS was significantly longer when patients had a good ECOG (0-1). This agrees with the results in the TRUST study [12]. In this study, a correlation between a long median PFS and high grades of rash was also mentioned and was observed in our study.

In general, erlotinib was well tolerated. Side effects were mostly rash and diarrhoea, which were well manageable via symptomatic therapy, dose reductions, and/or interruption of therapy. Regarding all therapy lines, no significant differences in the adverse events were observed. Patients with third-line erlotinib therapy had the lowest rate of severe side effects, but as this phenomenon is not yet described in larger studies, it may be the result of a small sample size.

The third-line group had the longest median PFS and simultaneously the slightest side effects. Nevertheless, median OS was shortest in this group—maybe because third-line patients already had longer survival from diagnosis compared to patients in the second-line group. The group undergoing fourth-line and more-line therapy had the highest DCR. It may be speculated that patients without EGFR-M may have the highest benefit from erlotinib in third-line and more-line therapy.

*Limitations*. The study was conducted retrospectively in only Caucasian patients and the sample size was relatively small. Nevertheless, there are only a limited number of patients receiving various therapy lines. Additionally, as the patients were involved in the decision to receive erlotinib or chemotherapy, they may be more compliant than usual. A further limitation is the monocentric study design, which may have caused bias. Nevertheless, this is a study in routine clinical practice without possible bias related to external funding.

## 5. Conclusion 

Erlotinib is an orally applicable, well-tolerated antitumour drug with the most common side effects being mild or moderate rash and diarrhoea. The median PFS of erlotinib therapy was 83 days, median OS was 7 months, and DCR was 66.2%. Patients with an EGFR mutation had the longest median PFS (204 days) and the highest response rate (33%). Erlotinib worked in all therapy lines without any significant differences in efficacy and side effects. Comparing therapy lines, erlotinib in third-line offered the longest median PFS, while patients with erlotinib in fourth-line and more-line therapy had the longest median OS, DCR, and 1-year survival rate. However, these results were not statistically significant. Therefore, the results should be proven in a large multicenter study.

## Figures and Tables

**Table 1:** Patients' baseline characteristics (*n* = 84).

Characteristics (*n* (%))	*n* = 84
Median age (years)	70 (range 50–84)
Sex	
Male	73 (86.9)
Female	11 (13.1)
ECOG PS∗	
0	2 (2.4)
1	40 (47.6)
2	35 (41.7)
3	7 (8.3)
Stage	
IIIB	2 (2.4)
IV	82 (97.6)
Histology	
Squamous cell carcinoma	47 (56.0)
Adenocarcinoma	20 (23.8)
NSCLC (NOS)	13 (15.5)
Large cell carcinoma	2 (2.4)
Adeno- and Squamous cell carcinoma	2 (2.4)
Prior lines of chemotherapy	
One	34 (40.5)
Two	36 (42.9)
Three	12 (14.3)
Four	1 (1.2)
Five	1 (1.2)
Smoking status at time of starting of erlotinib	
Never smoker	20 (23.8)
Current or former smoker	64 (76.2)
EGFR mutation status	
Positive	4 (4.8)
Negative	20 (23.8)
Unknown	60 (71.4)

ECOG PS∗: Eastern Cooperative Oncology Group (ECOG) performance status.

NOS: not otherwise specified.

**Table 2:** Progression-free survival (PFS), overall survival (OS), disease control rate (DCR), and one-year survival in different erlotinib therapy lines.

	All lines (*n* = 84)	Second-line (*n* = 34)	Third-line (*n* = 36)	Fourth-line and more-line (*n* = 14)	*P* value
PFS (days)	83 (CI 70.0–96.0)	80 (CI 67.0–93.0)	89 (CI 58.6–119.4)	84 (55.8–112.2)	0.886^1^
OS (months)	7 (CI 4.7–9.3)	7 (CI 4.1–9.9)	4 (CI 2.6–5.3)	9 (CI 1.34–16.7)	0.178^1^
DCR	66.2% (CI 54.3%–76.8%)	58.6% (CI 38.9%–76.5%)	69.7% (CI 51.3%–84.4%)	75% (CI 42.8%–94.5%)	0.542^2^
1-year survival rate	33% (CI 22%–43%)	31% (CI 14%–47%)	28% (CI 13%–43%)	49% (CI 22%–76%)	0.178^1^

^1^log rank test.

^2^Chi^2^ test.

**Table 3:** Most important adverse events in 75 patients with erlotinib therapy.

Event/therapy line	Second-line (*n* = 32)		Third-line (*n* = 30)		Fourth-line and more-line (*n* = 13)	
	Grades 1-2	Grades 3-4	Grades 1-2	Grades 3-4	Grades 1-2	Grades 3-4
All	17 (53.1%)	13 (40.6%)	16 (53.3%)	9 (30.0%)	7 (53.8%)	5 (38.5%)
Rash	16 (50.0%)	6 (18.8%)	16 (53.3%)	5 (16.7%)	7 (53.8%)	2 (15.4%)
Diarrhoea	11 (34.4%)	3 (9.4%)	8 (26.7%)	3 (10.0%)	4 (30.8%)	2 (15.4%)
Emesis	2 (6.3%)	1 (3.1%)	4 (13.3%)	1 (3.3%)	3 (23.1%)	2 (15.4%)
Dyspnea	4 (12.5%)	2 (6.3%)	3 (10.0%)	0 (0%)	1 (7.7%)	1 (7.7%)
Pain	1 (3.1%)	2 (6.3%)	3 (10.0%)	1 (3.3%)	0 (0%)	1 (7.7%)
Neurotoxicity	4 (12.5%)	0 (0%)	2 (6.7%)	0 (0%)	1 (7.7%)	1 (7.7%)
Stomatitis	4 (12.5%)	0 (0%)	2 (6.7%)	0 (0%)	1 (7.7%)	0 (0%)
Alopecia	3 (9.4%)	0 (0%)	2 (6.7%)	1 (3.3%)	1 (7.7%)	0 (0%)
Loss of appetite	1 (3.1%)	1 (3.1%)	4 (13.3%)	1 (3.3%)	0 (0%)	0 (0%)

## Abbreviations

CI: 95% confidence interval
CT: Computed tomography
DCR: Disease control rate
ECOG: Eastern Cooperative Oncology Group performance status
EGFR: Epidermal growth factor receptor
EGFR-M: Epidermal growth factor receptor mutation
HR: Hazard ratio
IQR: Interquartile range
NSCLC: Non-small-cell lung cancer
OS: Overall survival
PD: Progressive disease
PFS: Progression-free survival
PET: Positron emission tomography
PS: Performance status
PR: Partial response
SD: Stable disease
TKI: Tyrosine kinase inhibitor.
